# How do women and men differ in research collaborations based on authorship positions? The Spanish case

**DOI:** 10.3389/frma.2025.1631931

**Published:** 2025-08-13

**Authors:** Fernanda Morillo, Manuel Escabias, Zaida Chinchilla-Rodríguez

**Affiliations:** ^1^Instituto de Filosofía (IFS), Consejo Superior de Investigaciones Científicas (CSIC), Madrid, Spain; ^2^Department of Statistics and Operations Research, University of Granada, Granada, Spain; ^3^Instituto de Políticas y Bienes Públicos (IPP), Consejo Superior de Investigaciones Científicas (CSIC), Madrid, Spain

**Keywords:** gender disparities, authorship position, research collaboration, research assessment, publishing career stage, international collaboration, industry collaboration

## Abstract

This study examines gender disparities in authorship and collaboration within the Spanish scientific workforce, focusing on international and industry co-authored publications. Drawing on a comprehensive dataset of over 165,000 publications and more than 170,000 identified authors affiliated with Spanish institutions, the analysis explores how gender interacts with authorship position, research field, career stage, and team size. The results reveal a consistent under-representation of women in both types of collaboration, particularly in key authorship roles (first, last, and corresponding author). While women are more active at early career stages, their visibility in leadership roles tends to diminish over time, especially as the number of co-authors increases. Field-specific patterns show that even in highly feminized disciplines, such as Biomedical & Health Sciences, women are less likely to appear in prominent authorship positions. These findings raise important concerns about current research assessment practices that rely heavily on byline position as a proxy for contribution or leadership. The study contributes to ongoing discussions on responsible metrics and proposes policy recommendations to promote more equitable evaluation systems that reflect the collaborative and diverse nature of research careers.

## 1 Introduction

Science policy bodies worldwide recognize and actively promote the importance of collaboration in research. For instance, the HORIZON Europe program supports EU Member States in harnessing their national research and innovation potential. It aims to foster closer collaborations while strengthening technological and industrial capacities (HORIZON EUROPE, 2020). Similarly, the Spanish State Plan for Scientific and Technical Research and Innovation (Spanish State Plan of Scientific and Technical Research and Innovation, 2024) encourages collaboration and bolsters international leadership through thematic, sectoral, and territorial synergies, alongside promoting public-private partnerships. These collaborations often involve an increasing number of authors and cross-institutional partnerships, all aimed at addressing local and complex challenges (Wang and Barabási, 2020).

However, despite policy efforts that have increasingly promoted collaborative research as a driver of innovation and impact (Georghiou et al., 2014), research assessment practices tend to prioritize individual authorship roles, such as corresponding, first, or last author positions, or principal investigator status (Bu et al., 2020). This emphasis on individual contributions often outweighs recognition of the collaborative structures that make knowledge generation possible within research groups (Klein and Falk-Krzesinski, 2017). Moreover, previous studies based on the same dataset have shown that these authorship positions are directly linked to national evaluation requirements, particularly in research fields where gender disparities in participation are more pronounced (Chinchilla-Rodríguez et al., 2025), so that the benefits of collaboration are not always evenly distributed among all partners (Chinchilla-Rodríguez et al., 2019). Moreover, research collaboration patterns and assessment practices have been found to exhibit gendered dynamics (Fox, 2020), though findings vary across international (Kwiek and Roszka, 2021) and academic-industrial collaborations (Fell and König, 2016). Collaboration patterns also differ across research fields, with international cooperation being more prevalent in some disciplines than others. Women tend to be concentrated in fields related to health, education, and social sciences, and often specialize in topics that may influence their collaborative opportunities—both internationally and with industry (Haghani et al., 2022; Kwiek and Roszka, 2021). These disciplinary and thematic concentrations may partly explain the unequal distribution of benefits and visibility within collaborative settings. Given the pivotal role of collaboration in research, it is crucial to examine how it may differ systematically across various dimensions, including gender. This study seeks to explore potential gender differences in the distribution of the Spanish workforce in international and industry collaborations. The research adopts a comprehensive and contextualized perspective, considering not only gender but also factors such as authorship position, research field, academic age, and the number of co-authors involved.

## 2 Related work

Collaboration in science is fundamental to the research ecosystem. It enhances the scientific and technological capabilities of all partners and plays an important role in the social construction of science (Chinchilla-Rodríguez et al., 2016). On an international scale, scientific collaboration is driven by the pursuit of technological advancements and the need to remain competitive and innovative (Coccia and Wang, 2016). However, several factors shape how these collaborations unfold, particularly in the dynamics of knowledge creation (Wagner et al., 2015). For instance, the unequal flow of knowledge between industrialized and peripheral countries (Beigel and Hanan, 2014) and the differences across disciplines (Thelwall and Maflahi, 2020) influence collaboration patterns. Collaboration is generally more common in the natural sciences than in the humanities (Aksnes et al., 2019), and female participation in international collaborations is often limited (Kwiek and Roszka, 2021). Despite this, robust collaborative networks can improve women's academic performance (Zhang et al., 2020).

When looking at specific countries, the influence of gender on international collaboration can be less clear. For example, in countries like Norway, gender differences in foreign interactions become statistically insignificant when considering academic position, productivity, and scientific field (Aksnes et al., 2019). However, gender and productivity are complexly linked, with women typically being less prolific than men (González-Salmón et al., 2025). The situation varies by country: female researchers in Italy tend to collaborate more nationally than internationally (Abramo et al., 2019), while in India, female researchers produce more internationally collaborative articles, although their study only focused on first authors (Paswan and Singh, 2020).

Industrial collaborations are crucial for fostering innovation, and they are shaped by multiple factors, including academic incentives, organizational structures, and individual motivations (Perkmann et al., 2021). These partnerships can significantly impact researchers, but they often present challenges, such as requiring adaptation to external expectations (Bozeman and Gaughan, 2011). Incentives are frequently provided to encourage industry collaborations, though they may be offset by disincentives, such as transaction costs, which depend on team heterogeneity (Abramo and D'Angelo, 2022). Tartari and Salter (2015) highlight that women may experience career setbacks if they are less involved in industrial collaborations, as public sector research increasingly demands such partnerships to maximize social and economic impacts. Gender differences in collaboration strategies also emerge: male researchers are more likely to adopt a “mentoring” approach, while female researchers are often seen as more focused on academic and teaching roles (Bozeman and Gaughan, 2011; Huang et al., 2019).

Authorship order in academic publications plays a vital role in research assessments. The position of an author in the byline—particularly as first or last author—is often interpreted as an indicator of their contribution to the research. First authorship is typically associated with the greatest contribution (Larivière et al., 2016), while the last author position can signal seniority (Kuper et al., 2023). More recently, corresponding authorship has also been linked to leadership (Chinchilla-Rodríguez et al., 2024). Although authorship order can vary depending on academic practices (Lu et al., 2022), funding models, and institutional agreements (Zhang et al., 2022), it can influence team composition, Research Topics, and individual career advancement (Bozeman et al., 2013; Chinchilla-Rodríguez et al., 2024). These factors can lead to disputes over authorship positions (Fleming, 2021).

Gender disparities in academic publishing may further exacerbate the challenges women face in academia (Lundine et al., 2018). The underrepresentation of women in prestigious authorship positions can hinder their long-term academic careers, particularly as they advance to higher stages (Kwiek and Roszka, 2021; West et al., 2013). Furthermore, when research assessments overly emphasize byline positions, researchers—especially women—may be discouraged from engaging in other essential academic activities, such as teaching and mentoring (Hatch and Curry, 2020).

### 2.1 Purpose and objectives

In Spain, the National Agency for Quality Assessment and Accreditation (ANECA) is responsible for the evaluation, certification, and accreditation of the higher education system (ANECA, 2022). Recently, the evaluation criteria for recognizing research quality and academic accreditation have been reformed, following the agreements established in December 2022 by the Coalition for Advancing Research Assessment (CoARA, 2022).

This article aims to address gender disparities in the distribution of the Spanish research workforce involved in international and industry collaborative publications, through a more diverse and inclusive analysis of scientists' research careers. In this context, we understand *disparities* as systematic gender-based differences in the level of participation and leadership, particularly in key authorship positions (first, last, and corresponding author), which may reflect underlying inequalities in how scientific contributions are recognized and valued. Additional variables such as research field, career stage, and number of co-authors are considered.

This study is based on the following assumptions:

(1) Collaboration is not neutral in its design or outcomes. While it is widely promoted as a sign of research excellence and is increasingly incentivized through science policy and funding schemes, collaboration practices often reflect existing structural inequalities within the academic system—particularly regarding gender and seniority.
(2) Authorship position functions as a proxy for contribution and leadership. The positions of first, last, and corresponding author are commonly interpreted as indicators of scientific leadership, coordination, or intellectual ownership. These roles are often used in research assessment processes to infer individual merit and academic impact. However, access to such positions may be unequally distributed, especially in collaborative settings.
(3) International and industry collaborations differ institutionally but may share patterns of inequality. Although these two types of collaboration follow distinct institutional logics and purposes, both are considered strategic priorities in research policy and are often evaluated using similar criteria. Previous studies suggest that gender disparities are present in both, particularly in terms of access, recognition, and leadership. By analyzing them together, we can uncover shared structural patterns that limit visibility and advancement for under-represented groups, notably women.
(4) A multidimensional approach is needed to understand gendered dynamics in collaboration. Gender inequalities in science do not operate uniformly across fields, career stages, or team configurations. A comprehensive analysis must account for the interplay of these variables to identify where and how disparities emerge, and to inform more equitable evaluation practices.

Accordingly, this study seeks to answer several research questions, taking into account the different characteristics of the publications:

- Is there a gender disparity in the participation of women in both types of collaborative publications?
- How does the representation of women across career stages differ in international vs. industry collaborative publications? Are women under-represented in prominent authorship positions (first, last, or corresponding author)?
- Does the likelihood of women/men being corresponding authors decrease as the number of authors increases?
- What recommendations can be made to science policy-makers?

## 3 Material and methods

### 3.1 Data source and period of analysis

This study used the in-house version of the Web of Science (WoS) database maintained by the Centre for Science and Technology Studies (CWTS, Leiden University). This enriched dataset includes author name disambiguation, gender inference, academic age, and disciplinary classification. The period analyzed is 2015–2017, and all document types with at least one Spanish address were considered. This timeframe was selected because it corresponds to the most recent period with validated and complete bibliometric coverage available in the CWTS version of WoS.

The CWTS methodology for author identification was developed by Caron and van Eck (2014) and refined by D'Angelo and van Eck (2020), offering better performance than other approaches (Tekles and Bornmann, 2020). This method applies an unsupervised learning approach based on scoring and grouping rules. It considers author names by surname and initial, in conjunction with article, category, journal, and citation data, and then clusters authors according to the scores obtained. The process achieves a mean precision of 0.97 and a mean recall of 0.91 in author identification.

Meanwhile, gender identification is based on the author's name and country of origin. It uses three gender inference tools—Gender API, Gender Guesser, and Genderize.io—and applies several decision rules. This approach yields high precision: 95.8% for women and 97.6% for men (Boekhout et al., 2021). After applying this method, 14% of authors whose gender could not be clearly determined were excluded. The final dataset consists of 81,179 women (46%) and 95,574 men (54%), contributing to the authorship of 165,649 documents.

Each author was assigned to their respective byline positions in publications: first, middle, last, corresponding author, and combinations of first, middle, or last author as corresponding author. Collaboration is analyzed according to the number of co-authors and affiliation addresses. Two types of collaborative practices are distinguished: international collaborative publications, involving authors from at least two different countries, and industry collaborative publications, involving academic authors with at least one industrial partner. Note that a researcher may participate in both international and industry collaborations. Therefore, each researcher is counted in both types of collaboration where applicable, as participation is not mutually exclusive.

Academic age is calculated based on the years of the author's first and last publications, and classified into four categories: (a) 0–10 years, (b) 11–20 years, (c) 21–30 years, and (d) more than 30 years of publishing activity. Additionally, authors are classified into five main fields: Biomedical & Health Sciences (BIO), Life & Earth Sciences (LIF), Mathematics & Computer Science (MAT), Physics & Engineering (PHY), and Social Sciences & Humanities (SSH).

Finally, the Mann–Whitney *U*-test for independent samples is used to compare the distribution of prominent authorship positions by gender for each type of collaboration. Spearman's rank correlation coefficient is used to analyze the relationship between the number of co-authors and the likelihood of holding prominent positions. Results with a *p*-value below 0.05 are considered statistically significant.

## 4 Results

**Figure 1:**
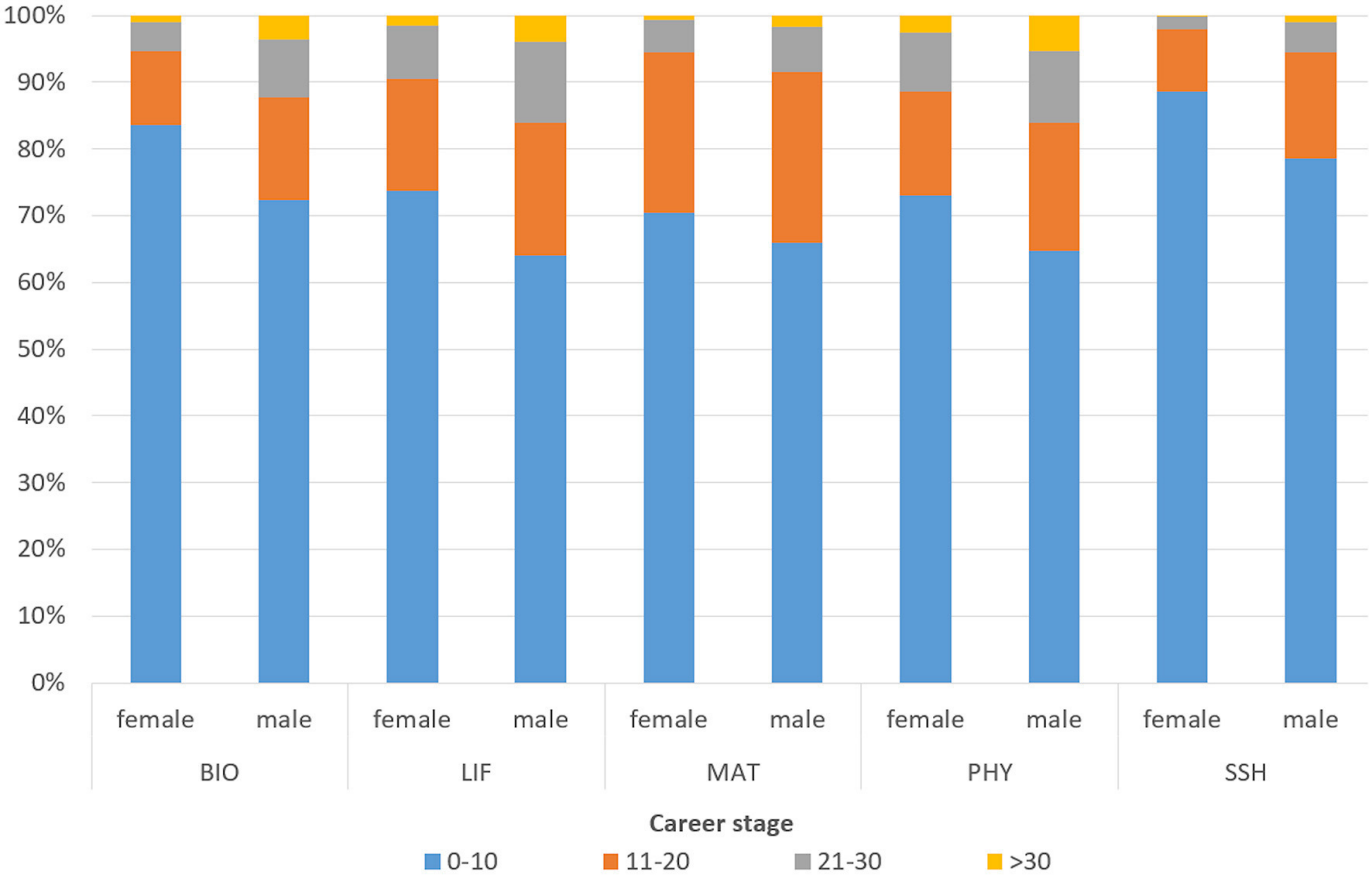
Distribution of authors by gender, research field and career stage. Career stages: 0–10 years (early), 11–20 years **(middle)**, 21–30 years (advanced), >30 years (consolidated). Research fields: Biomedical and Health Sciences (BIO), Life and Earth Sciences (LIF), Mathematics and Computer Science (MAT), Physics and Engineering (PHY), and Social Sciences and Humanities (SSH).

Figure 1 shows the distribution of the Spanish research workforce by gender, research field, and career stage to contextualize the analysis. Most researchers are concentrated in the early career stage (0–10 years), with the lowest percentages of men observed in LIF (64%) and PHY (65%), and the highest percentages of women in SSH (89%) and BIO (84%). These results are consistent with previous findings based on the same dataset, which show that the majority of women in the Spanish scientific workforce—especially in BIO, SSH, and LIF—are concentrated in early career stages, while their presence in senior cohorts is much more limited. Notably, in STEM fields such as MAT, women represent only a small fraction of consolidated researchers (Chinchilla-Rodríguez et al., 2025). MAT, which is the smallest field in terms of researcher count, has a lower overall share in the early stage but stands out for having one-quarter of its researchers in the intermediate stage (11–20 years) for both genders. Conversely, there is a notable shortage of researchers in the final career stage (>30 years), particularly among women in SSH and MAT. The highest relative presence of researchers in the final stage is found among men in PHY and LIF.

**Figure 2:**
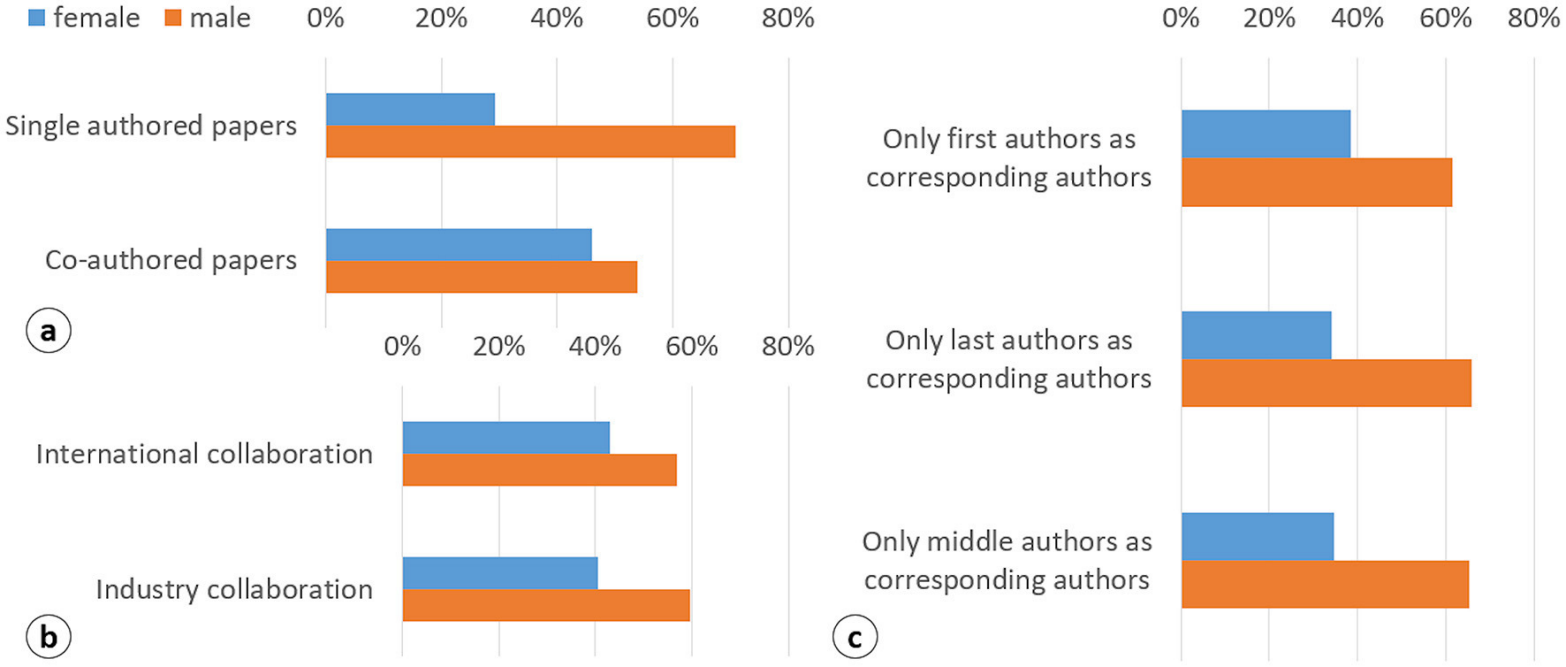
Distribution of authors by gender and collaboration type **(a, b)** and corresponding authorship positions in co-authored papers **(c)**. Gender distribution of authors in collaborative publications. **(a)** Overall share of male and female authors in co-authored papers. **(b)** Gender distribution in international and industry collaborations. **(c)** Proportion of male and female corresponding authors by byline position (first, middle, last). Percentages are calculated relative to the total number of authors in each category.

When analyzing all documents across fields and career stages, the results show that women in Spain are less likely than men to publish single-author articles (29% vs. 71%, respectively). As a result, the proportion of female co-authors is higher and closely mirrors the overall distribution of publications (46% women vs. 54% men; Figure 2a). In certain types of collaboration, the percentage of female participation decreases, particularly in industry collaboration (40%; Figure 2b). This percentage drops even further for corresponding authorship, where women are under-represented across all byline positions, as shown in Figure 2c.

**Figure 3:**
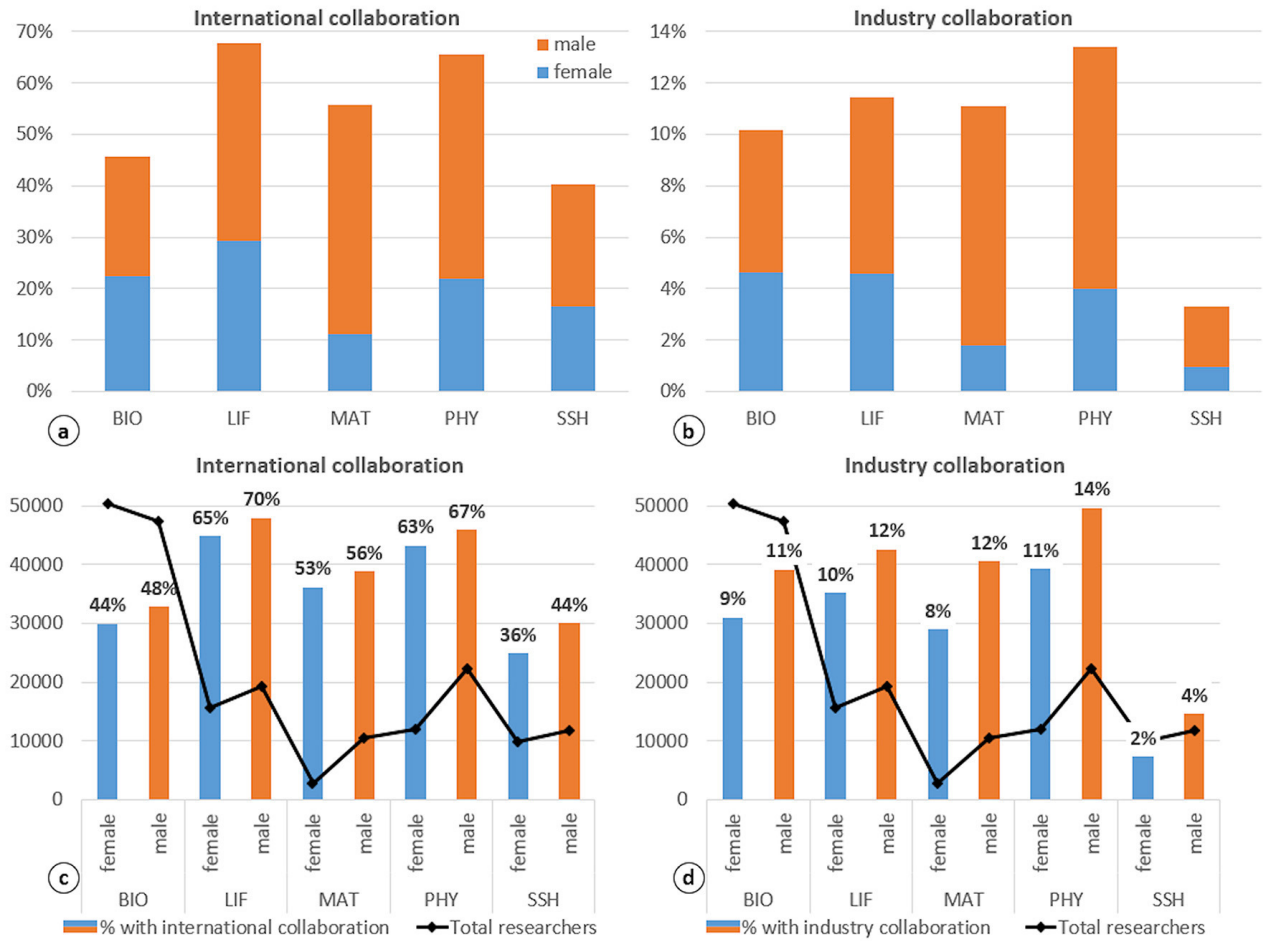
Distribution of authors by gender, collaboration type, and research field in proportion to the total of each field **(a, b)** and to the total of the analyzed publications **(c, d)**. In **(c, d)**, the bars represent the percentage of women (blue) and men (orange) relative to the total number of researchers (black line) in each type of collaboration. Research fields: Biomedical and Health Sciences (BIO), Life and Earth Sciences (LIF), Mathematics and Computer Science (MAT), Physics and Engineering (PHY), and Social Sciences and Humanities (SSH).

Figures 3a, b show the distribution of authors by gender, collaboration type (international and industry), and research field, in proportion to the total number of researchers in each field. Figures 3c, d present the same data but calculated in proportion to the total number of women and men in the analyzed publications. As shown in Figure 3a, LIF (68%) and PHY (65%) are the fields with the highest levels of international collaboration, while BIO and LIF have the largest proportions of women. These two fields also show the highest female participation in industry collaboration (Figure 3b), although PHY (13%) stands out as the field with the largest share of researchers involved in industrial collaboration. Looking at the total number of authors per research field (black line), Figures 3c, d show that there are fewer women than men in all fields except BIO (50,356 women and 47,333 men). MAT and PHY show the lowest proportion of female researchers, although both have a non-negligible share of international collaboration relative to the total number of women (Figure 3c). LIF is the field with the highest proportion of women (65%), whereas SSH has the lowest (36%). In terms of industry collaboration, PHY is the field with the highest proportion of women (11%) relative to the total number of female researchers (Figure 3d). Conversely, SSH is the field with the lowest proportion (2%), which is half that of men, despite the relatively balanced gender distribution in this field.

**Figure 4:**
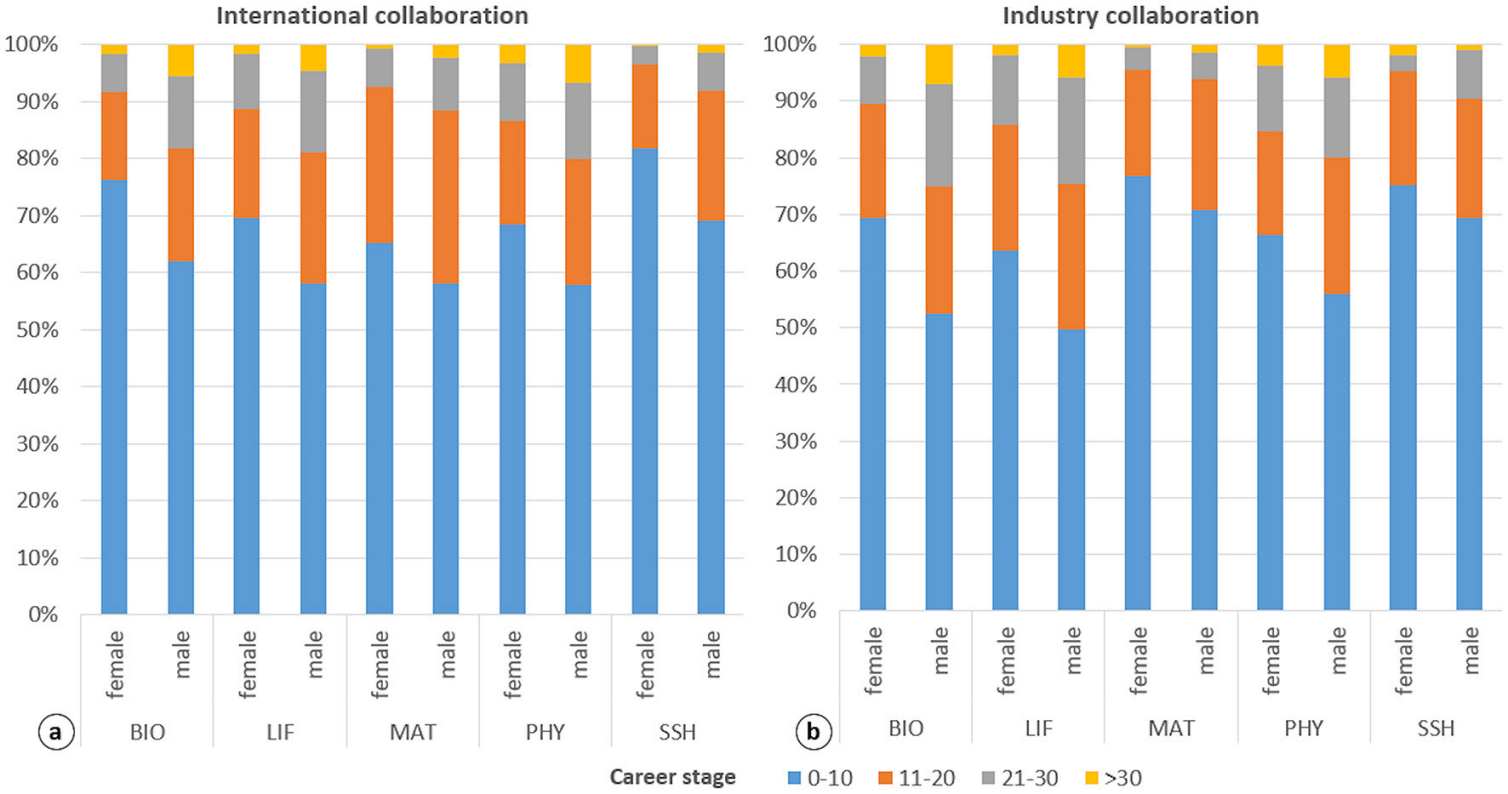
Distribution of authors by gender, collaboration type, research field and career stage. Career stages: 0–10 years (early), 11–20 years **(middle)**, 21–30 years (advanced), >30 years (consolidated). Research fields: Biomedical and Health Sciences (BIO), Life and Earth Sciences (LIF), Mathematics and Computer Science (MAT), Physics and Engineering (PHY), and Social Sciences and Humanities (SSH).

Figures 4a, b show that collaborative publications involving international or industry institutions are predominantly concentrated in the early stages of researchers' careers—an observation consistent with the overall pattern in the Spanish research workforce (see Figure 1). In international collaboration (Figure 4a), the highest female representation at the early career stage is observed in SSH (82%) and BIO (76%). By contrast, MAT shows the lowest share of early-career female participants (65%). Interestingly, MAT also has a notable proportion of researchers in the intermediate stage (27% women and 30% men). In contrast, LIF and PHY show higher shares of researchers in the most advanced stages of their careers (10% women and 14% men, respectively).

In industry collaborations (Figure 4b), the distribution is less skewed toward early-career researchers compared to international collaboration. MAT again shows the highest female representation at early stages (77%), followed closely by SSH (75%). Industry collaborations are more common at later stages of researchers' careers, particularly in LIF (intermediate stage) and PHY (consolidated stage). Notably, although the presence of women in these collaborations remains modest, they are proportionally better represented than men at the final stage in SSH (2%).

### 4.1 International collaboration

**Figure 5:**
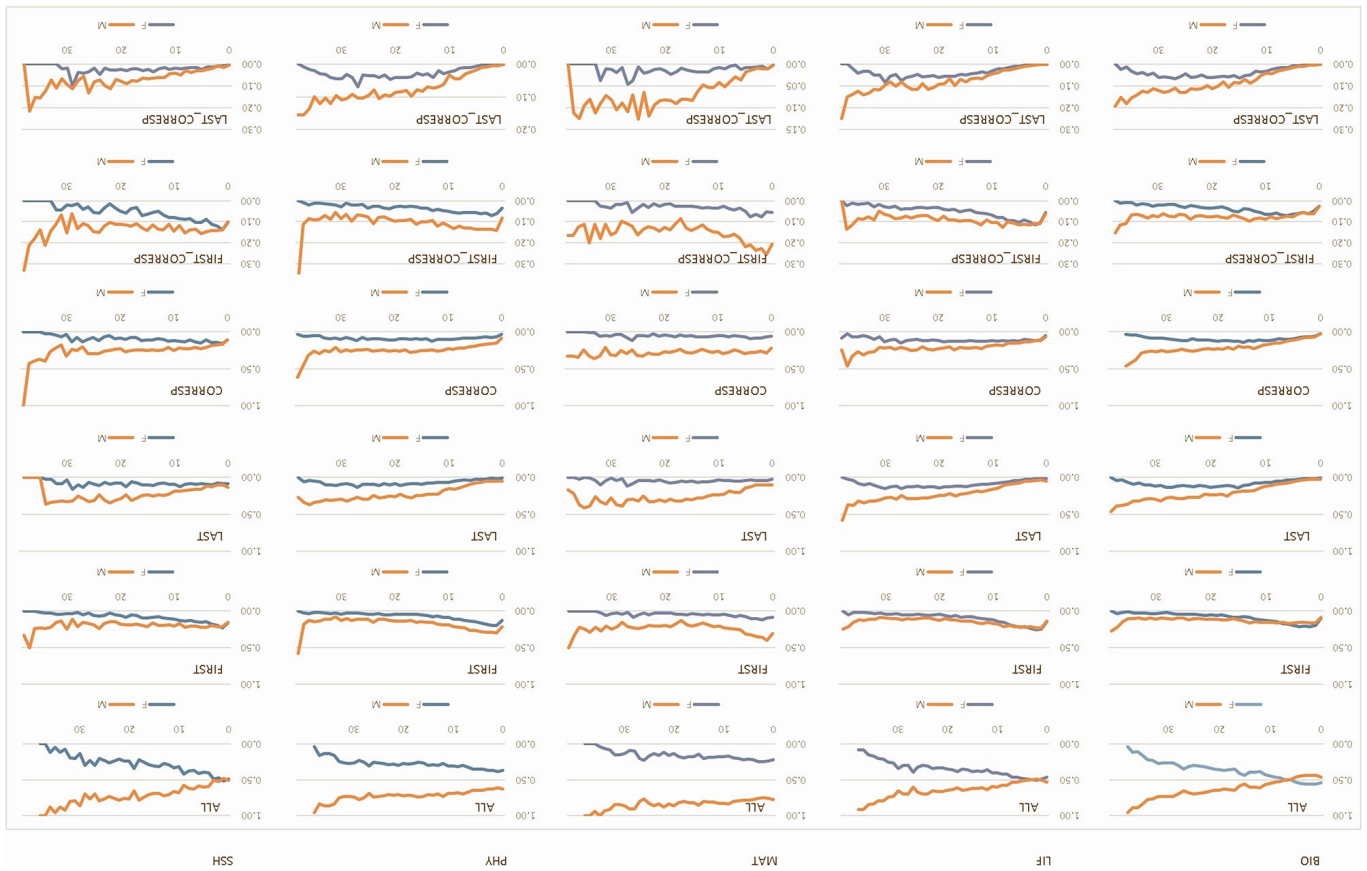
Probabilities of authors by gender, research field, and career stage in different positions in international collaboration. Probabilities are plotted on the vertical axis, while career stages are plotted on the horizontal axis. Gender: female (F) and male (M). Research fields: Biomedical and Health Sciences (BIO), Life and Earth Sciences (LIF), Mathematics and Computer Science (MAT), Physics and Engineering (PHY), and Social Sciences and Humanities (SSH). Career stages: 0–10 years (early), 11–20 years **(middle)**, 21–30 years (advanced), >30 years (consolidated). Byline positions: ALL, FIRST author, LAST author, CORRESP (corresponding author), FIRST_CORRESP (first author as corresponding author), LAST_CORRESP (last author as corresponding author).

Having established that men outnumber women across all research fields and career stages (see Figure 3), we now examine additional characteristics of international collaboration. Figure 5 presents the probability of women and men participating in international collaborative publications, disaggregated by authorship position (first, last, corresponding), research field, and career stage. The probability values range from 0 (impossible) to 1 (certain), with the total distribution summing to 1 per group.

Across all research fields, the probability of women participating in international collaborations tends to decline as their careers advance, while the opposite trend is observed for men. At early career stages, female researchers are most likely to appear as first authors, particularly in BIO and LIF, where their representation surpasses that of men. For men, the probability of first authorship increases progressively, especially in PHY, SSH, and (MAT).

The last author position is rarely held by either gender early in their careers. However, the probability for women increases slightly in mid-career stages, particularly in BIO and LIF, while it rises more substantially for men over time. Similar patterns are seen in the corresponding author role, where women are consistently under-represented. Notable exceptions include SSH, where women show a relatively strong early-career presence, and LIF and BIO, where some improvement is observed in mid and advanced career stages. Nevertheless, men's probability of corresponding authorship increases consistently with age in most fields, except MAT.

When considering combined positions—first/corresponding and last/corresponding author—women are more likely to hold the first/corresponding role in early stages, particularly in SSH and LIF. Trends for the last/corresponding position follow the patterns observed for last authorship.

Overall, women are significantly under-represented in key authorship positions (first, last, corresponding) in international collaborations (*p* < 0.05, Mann–Whitney *U*-test).

**Figure 6:**
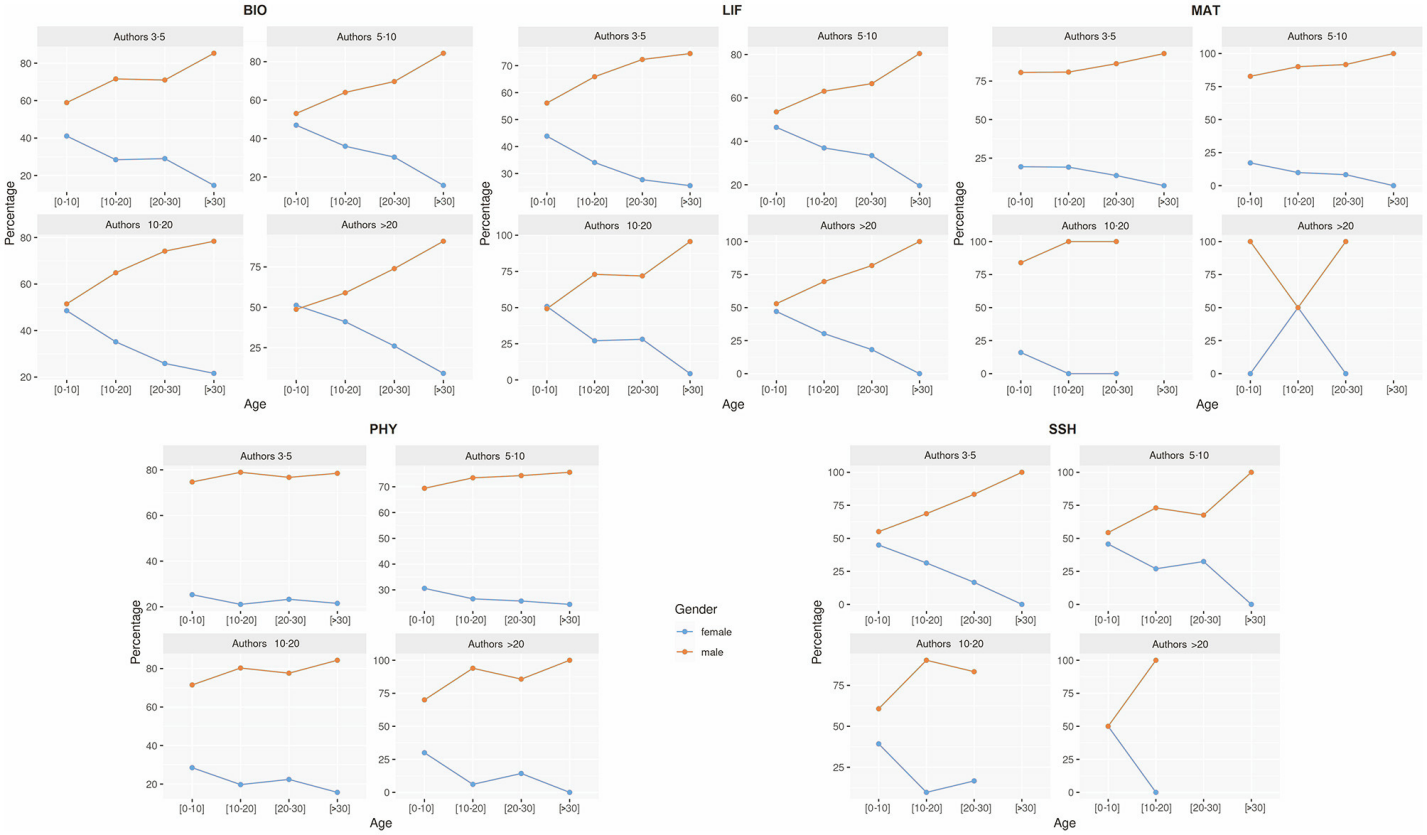
Distribution of corresponding authors by gender, research field, career stage, and number of co-authors in international collaboration. Research fields: Biomedical and Health Sciences (BIO), Life and Earth Sciences (LIF), Mathematics and Computer Science (MAT), Physics and Engineering (PHY), and Social Sciences and Humanities (SSH). Career stages: 0–10 years (early), 11–20 years **(middle)**, 21–30 years (advanced), >30 years (consolidated). Number of authors: 3–5, 5–10, 10–20, >20.

To further explore this dynamic, Figure 6 analyzes the relationship between corresponding authorship and the number of co-authors, by gender, research field, and career stage. In general, an increase in team size is associated with a lower likelihood of occupying relevant authorship positions (*p* < 0.05, Spearman's rank correlation). However, notable exceptions emerge. In BIO, for instance, women account for up to 51% of corresponding authorships in publications with more than 20 authors—a proportion that challenges the typical trend. A similar pattern is observed in LIF. In SSH, near gender parity is observed during early career stages, except in publications with 10–20 authors, where women are less represented. In contrast, MAT and PHY show consistently low female representation across all stages and team sizes.

### 4.2 Industry collaboration

**Figure 7:**
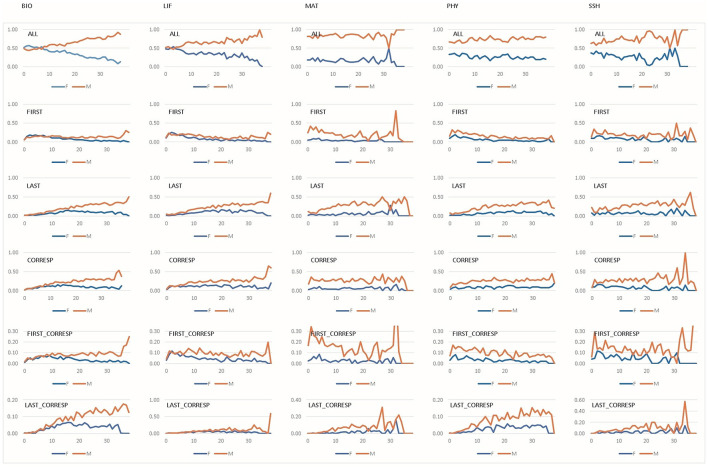
Probabilities of authors by gender, research field, and career stage in different positions in industry collaboration. Probabilities are plotted on the vertical axis, while career stages are plotted on the horizontal axis. Gender: female (F) and male (M). Research fields: Biomedical and Health Sciences (BIO), Life and Earth Sciences (LIF), Mathematics and Computer Science (MAT), Physics and Engineering (PHY), and Social Sciences and Humanities (SSH). Career stages: 0–10 years (early), 11–20 years **(middle)**, 21–30 years (advanced), >30 years (consolidated). Byline positions: ALL, FIRST author, LAST author, CORRESP (corresponding author), FIRST_CORRESP (first author as corresponding author), LAST_CORRESP (last author as corresponding author).

This section explores the characteristics of collaborative publications involving industry, where, as previously noted (Figure 3), women are even less represented than in international collaborations. Figure 7 illustrates the probability of researchers participating in such collaborations by gender, career stage, and byline position.

As with international collaboration, women are more likely to participate during the early stages of their careers and are most strongly represented in BIO. At this stage, the highest probabilities of holding the first author position are found among women in BIO and LIF. For men, the probability of first authorship peaks later in the career, particularly in MAT.

The probability of being the last author is generally lowest during the early stages. For women, it increases moderately during mid and advanced career stages, especially in LIF and BIO, and even surpasses that of men in MAT at the start of the final stage. For men, the probability of last authorship tends to rise over time, although this pattern is less clear in MAT and SSH.

In terms of corresponding authorship, women display the lowest probabilities overall, particularly in early career stages. Slight increases are observed during the middle and advanced stages, followed by a decrease and a final-stage peak in most fields—except in MAT and SSH, where their presence remains consistently low. As seen in international collaborations, women are more likely to be first and corresponding authors at early stages, especially in LIF and SSH, whereas men show more fluctuation, particularly in MAT and SSH. The probability of being a last and corresponding author increases with career progression for both genders. For women, this trend is most visible in BIO (middle career stage), MAT, and SSH (advanced stages). For men, late-career peaks are also observed in MAT and SSH.

Overall, women are significantly under-represented in last authorship positions and corresponding authorship roles in industry collaborations (*p* < 0.05, Mann–Whitney *U*-test).

**Figure 8:**
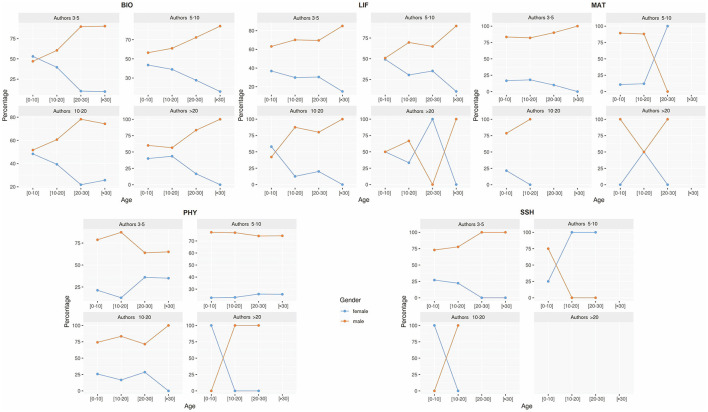
Distribution of corresponding authors by gender, research field, career stage, and number of co-authors in industry collaboration. Research fields: Biomedical and Health Sciences (BIO), Life and Earth Sciences (LIF), Mathematics and Computer Science (MAT), Physics and Engineering (PHY), and Social Sciences and Humanities (SSH). Career stages: 0–10 years (early), 11–20 years **(middle)**, 21–30 years (advanced), >30 years (consolidated). Number of authors: 3–5, 5–10, 10–20, >20.

To better understand these patterns, Figure 8 analyzes the relationship between corresponding authorship and the number of co-authors. As in international collaborations, an increase in the number of co-authors is associated with a lower probability of holding key authorship positions (*p* < 0.05, Spearman's rank test). Notably, in early career stages, women surpass men in some configurations—most prominently in LIF, where they account for 58% of corresponding authorships in publications with 10–20 authors. Conversely, in the later stages of their careers, women often disappear entirely from many co-authorship scenarios.

There are also several striking instances of 100% female representation in corresponding authorship across different fields, team sizes, and career stages—including in LIF, MAT, PHY, and SSH—despite the overall low presence of women in these collaborations.

## 5 Discussion and conclusions

This study provides a comprehensive examination of gender disparities in Spanish scientific collaboration, focusing on two key types—international and industry co-authorships—and analyzing them across research fields, academic age, author positions, and team size. Although these collaboration types are not inherently complementary, they reveal parallel patterns of gender inequality, raising important implications for science policy and research evaluation.

Persistent gender imbalances characterize both collaboration types. Women remain under-represented, especially in MAT and PHY, which confirms prior findings (Kwiek and Roszka, 2021). Interestingly, when we consider the share of female participation relative to the total number of women researchers, fields like MAT and PHY show unexpectedly high levels of international engagement. This suggests a qualitative involvement that contrasts with their overall under-representation. However, even in feminized fields such as BIO and SSH, women do not achieve parity in collaborative outputs.

Career stage plays a critical role in shaping collaboration dynamics. While gender gaps in science are widely documented in terms of representation, productivity, and impact, less attention has been paid to how these disparities manifest in collaborative practices across career stages. This is particularly relevant as the transition from researcher to research leader is still not well understood (Browning et al., 2017). In both international and industry contexts, female participation peaks at early career stages, aligning with broader workforce trends. Yet in later stages—where strategic positions and career-defining roles often concentrate—women's representation drops significantly. A few exceptions, such as female last authorship in MAT during the final stage of industry collaboration, underscore the complex, field-specific trajectories of women in science.

Gender disparities are especially pronounced in key authorship positions. In both collaboration types, women are less likely to appear as first, last, or corresponding authors, with statistically significant differences (*p* < 0.05), except for first authorship in industry collaboration. These positions are often interpreted as indicators of leadership or intellectual contribution and are crucial in research evaluation (Jappelli et al., 2017). While some mid-career gains are observed in BIO and LIF, these improvements are rarely sustained at later stages.

The number of co-authors also influences gender dynamics. Larger team sizes generally correspond to a lower probability of women holding prominent positions. However, exceptions in BIO and LIF—where women maintain or even increase their presence in large international teams—highlight that team composition and field-specific cultures can modulate these effects. Moreover, isolated cases of 100% female corresponding authorship across diverse fields and stages reflect both the progress made and the fragility of these gains.

These findings challenge the assumption that collaborative science is inherently equitable. On the contrary, they suggest that without intentional policy interventions, collaboration may amplify existing gender disparities, particularly in how credit and recognition are distributed.

To address this, science policy-makers must move beyond one-size-fits-all approaches. Evaluation systems that rely heavily on byline positions risk overlooking contributions from women and early-career researchers, thereby reinforcing structural barriers. Institutions should adopt clear and inclusive authorship guidelines, encourage transparency through contributorship models, and ensure that collaboration is rewarded not just in terms of output but also in equitable role distribution.

Additionally, targeted support is needed in fields where women face compounded challenges—such as “double ghetto” environments in MAT and PHY (Tartari and Salter, 2015). Interventions such as early mentoring, inclusive leadership training, and gender-sensitive funding schemes can help retain diverse talent and counteract attrition during critical career transitions.

The findings of this study, part of the RESPONSIBLE project, offer evidence-based insights for reforming research evaluation systems under the principles of responsible metrics. By integrating dimensions such as gender, discipline, and career stage, institutions can build fairer assessment practices that value diversity, collaboration, and sustained contributions throughout a researcher's career.

Finally, we acknowledge some limitations, including the restricted time frame, reliance on a single data source, and the use of a national classification of career stages that is currently under review. Future research will explore open-access datasets, harmonize career stage definitions at the European level, and expand the use of contributorship frameworks to better capture collaborative contributions beyond byline order.

## Data Availability

The data analyzed in this study is subject to the following licenses/restrictions: The data analyzed in this study were obtained from the in-house version of the Web of Science database maintained by the Centre for Science and Technology Studies (CWTS, Leiden University). Due to licensing restrictions, these data cannot be shared publicly. Access to the dataset requires permission from CWTS and adherence to the terms of their data agreements. Requests to access these datasets should be directed to zaida.chinchilla@csic.es.
